# Commentary: SARS-CoV-2 Cell Entry Depends on ACE2 and TMPRSS2 and Is Blocked by a Clinically Proven Protease Inhibitor

**DOI:** 10.3389/fonc.2020.01448

**Published:** 2020-08-19

**Authors:** Alexandra Lindsey Zune Djomkam, Charles Ochieng' Olwal, Theodore Beyeme Sala, Lily Paemka

**Affiliations:** ^1^West African Centre for Cell Biology of Infectious Pathogens (WACCBIP), University of Ghana, Accra, Ghana; ^2^Department of Biochemistry, Cell and Molecular Biology, College of Basic and Applied Sciences, University of Ghana, Accra, Ghana; ^3^Department of Urology, Laquintinie Hospital, Douala, Cameroon

**Keywords:** coronavirus, SARS-CoV-2, COVID-19, prostate cancer, ACE2, TMPRSS2

In December 2019, a novel pneumonia condition termed coronavirus disease 2019 (COVID-19) was reported in Wuhan, China (1). The global burden of COVID-19 is increasing exponentially and as of 2nd July 2020, there were over 10,834,240 confirmed cases in about 213 countries and territories, with more than 519,590 fatalities (https://www.worldometers.info/coronavirus/). Currently, there are no specific antivirals or vaccines approved against COVID-19.

COVID-19 is caused by severe acute respiratory syndrome coronavirus 2 (SARS-CoV-2), a new member of coronaviruses, a group of enveloped, positive-sense, single-stranded RNA viruses (2). SARS-CoV-2 likely originated from *Rhinolophus affinis* bat species, based on 96.2% nucleotide sequence identity to the bat coronavirus, BatCoV RaTG13 (3). The virus causes more severe disease in males than in females. Furthermore, COVID-19 is more severe in older persons than the youth (4). It is largely unclear why there is differential severity in age and sex. However, the severity of COVID-19 in males could be related to their higher ACE2 profiles (5).

Here, leveraging the recent finding by Hoffmann et al. (1) that ACE2 and TMPRSS2 are critical for SARS-CoV-2 cell entry, we discuss the potential link between the SARS-CoV-2 receptors and the prostate gland and whether SARS-CoV-2 is a potential oncogenic virus for prostate cancer.

## SARS-CoV-2 Entry Into Human Cells

Entry of coronaviruses into target cells is facilitated by the spike (S) protein. Entry is dependent on binding of the surface unit, S1, of the S protein to a cellular receptor, which enhances viral attachment to the surface of target cells. Entry also requires priming of S protein by cellular proteases. The priming involves S protein cleavage at the S1/S2 and the S2' site to allow viral and cellular membrane fusion, a process driven by the S2 subunit. SARS-CoV-2 engages angiotensin-converting enzyme 2 (ACE2) as the entry receptor and serine protease TMPRSS2 for the S protein priming (1). The primary physiological role of ACE2 is the maturation of angiotensin, a peptide hormone that regulates vasoconstriction and blood pressure (2). ACE2 is a type I membrane protein expressed in the heart, lungs, kidneys, testes and intestine (2, 4). Reduced expression of ACE2 is linked with many chronic conditions (2). Other conditions that are exacerbated by high ACE activity, such as prostate cancer (6), are potentially affected by SARS-CoV-2 infection, which reduces ACE2 since reduced ACE2 implies upregulated ACE activity (4).

On the other hand, TMPRSS2 plays a major role in SARS-CoV-2 cell entry and is coincidentally dysregulated in prostate cancer. Additionally, TMPRSS2 is highly expressed in prostate epithelial cells in an androgen-dependent manner. Taken together, the findings that SARS-CoV-2 utilizes ACE2 and TMPRSS2 presupposes that SARS-CoV-2 could be an oncogenic virus for prostate cancer. For cells lacking or with reduced TMPRSS2 expression, Furin preactivation promotes SARS-CoV-2 entry into target cells (7). Subsequently, we discuss why SARS-CoV-2 could be linked with prostate carcinogenesis.

## Interplay Between SARS-CoV-2 Cell Entry Molecules and Prostate Biology

The role of renin-angiotensin system (RAS) and ACE in the pathology of carcinomas is well-established. ACE generates an effector peptide of the RAS system, angiotensin II (Ang II) via degradation of the vasodilator kinins (8). The prostate independently synthesizes ACE. The angiotensin II type 1 receptor is the predominant Ang II prostatic receptor. Inhibition of ACE activity has been shown to suppress tumor growth and angiogenesis *in vitro* and *in vivo* in animal models (9). Considering that ACE2 antagonizes the effects of ACE, it is probable that downregulation of ACE2 expression as in SARS-CoV-2 infection (4), which implies elevated ACE activity, may potentiate prostate carcinogenesis. This view is fortified by recent observation of SARS-CoV 2 in the semen of COVID-19 patients (10). The observation suggests that the virus could infect the prostate gland via SARS-CoV-2 entry molecules (ACE2 and TMPRSS2 or Furin), which are expressed by the prostate cells (11, 12).

TMPRSS2 protein, utilized by SARS-CoV-2 for S protein priming is highly expressed in normal prostate epithelial and prostate cancer cells. Moreover, TMPRSS2 is expressed in an androgen-dependent manner, particularly in the prostate (13). Additionally, the fact that signaling through the androgen receptor (AR) axis facilitates prostate cancer development (14), suggests a relationship between TMPRSS2 and prostate cancer. Studies have linked TMPRSS2 to prostate cancer via a chromosomal translocation resulting in the fusion of the TMPRSS2 promoter-enhancer with the Erythroblast Transformation Specific (ETS) transcription factors ETS-related gene (*ERG*) and ETS translocation variant 1 (*ETV1*) (13). This fusion recruits AR and TOP2B topoisomerase to chromosomal sites, where TOP2B instigates double-stranded breaks in DNA. Indeed, TMPRSS2-ERG fusion is associated with 40–70% of prostate cancer (13). SARS-CoV-2, cell entry is expected to reduce TMPRSS2, hence, lowering the TMPRSS2-ERG fusion. However, TMPRSS2 expression is increased in cells adjacent to SARS-CoV-2-infected cells (15). The increased TMPRSS2 expression could then promote TMPRSS2-ERG fusion events, hence predisposing male SARS-CoV-2-infected patients to prostate cancer. Alternatively, for cells lacking or with reduced TMPRSS2 expression, Furin preactivation promotes SARS-CoV-2 entry into target cells (7). Notably, the prostate gland and prostate cancer cells express Furin (11); thus, the virus may efficiently enter the prostate gland and/or prostate cancer cells using TMPRSS2 or Furin to initiate or enhance carcinogenesis.

## Is SARS-CoV-2 a Potential Oncogenic Virus for Prostate Cancer?

Is SARS-CoV-2 therefore an oncogenic virus? First, the expression of SARS-CoV-2 cell entry molecules in the prostate gland strongly suggests a SARS-CoV-2 prostate gland tropism. Secondly, SARS-CoV-2 infection is characterized by chronic inflammation (16). Chronic inflammation causes aberrant DNA methylation, which promotes cancer development (17). Inflammation is linked to about 60% of prostate cancer cases (18).

Changes in expression levels of TMPRSS2 have been previously associated with prostate cancer independently of SARS-CoV-2. Elevated expression of TMPRSS2 in the context of SARS-CoV-2 infections (15) implies that SARS-CoV-2 infection could increase chances of TMPRSS2 fusions, a phenomenon well-associated with prostate cancer development and progression (13). It is not precisely clear how SARS-CoV-2 may induce prostate cancer considering that it has not been shown to encode any known oncoprotein. However, given that SARS-CoV-2 can infect prostate cells, at least theoretically, it is probable that the virus can prompt prostate carcinogenesis via modulation of TMPRSS2 and/or exacerbating chronic inflammation in SARS-CoV-2 infected males (16). Taken together, it is plausible to hypothesize that SARS-CoV-2 could be an oncogenic virus for prostate cancer.

## Conclusions

SARS-CoV-2 and other coronaviruses are likely to remain in our midst for a long time. Although efforts are now geared toward the immediate preventive and treatment measures to avert fatalities, what we have presented above suggests that SARS-CoV-2 and similar coronaviruses could have long term effects such as involvement in cancer development. Specifically, we have highlighted a possible link between SARS-CoV-2 and prostate cancer based on the involvement of SARS-CoV-2 cell entry molecules on prostate biology. Nevertheless, detailed molecular and cell biology studies are warranted to prove our hypotheses.

## Author Contributions

AD and CO conceived and prepared the first draft of the manuscript. TS and LP critically reviewed the draft. All authors approved the final version of the manuscript.

## Conflict of Interest

The authors declare that the research was conducted in the absence of any commercial or financial relationships that could be construed as a potential conflict of interest.
